# A Novel Circularly Polarized Folded Transmitarray Antenna with Integrated Radiation and Scattering Performance

**DOI:** 10.3390/s22155503

**Published:** 2022-07-23

**Authors:** Zexu Guo, Xiangyu Cao, Tao Liu, Jun Gao, Sijia Li, Huanhuan Yang, Tong Li

**Affiliations:** Information and Navigation College, Air Force Engineering University, Xi’an 710077, China; zexuguo001@163.com (Z.G.); lt9571@163.com (T.L.); gjgj9694@163.com (J.G.); lsj051@126.com (S.L.); jianye8901@126.com (H.Y.); surgeonli4@163.com (T.L.)

**Keywords:** high-gain antenna, low RCS, axis ratio bandwidth, beam pattern

## Abstract

In this paper, a novel design method of circularly polarized folded transmitarray antenna (CPFTA) is presented by applying sequential rotation technology. Compared with the general design method, the novel design method can reduce the design difficulty and improve the axis ratio (AR) bandwidth significantly without adding any additional structure. To verify the proposed method, both a general CPFTA (GCPFTA) and a novel CPFTA (NCPFTA) are designed, fabricated, measured and compared. Good agreements between simulated and measured results are obtained. Thanks to the creative design, the integrated radiation and scattering control of the CPFTA is realized for the first time. The proposed NCPFTA has the advantages of broadband, high gain, planar structure, low profile, convenience in installation and low radar cross section (RCS), which has potential application in mobile satellite communication.

## 1. Introduction

Metasurfaces play an important role in the manipulation of amplitude [1,2] phase [3,4] and polarization [5,6] of electromagnetic wave. Reflectarray and transmitarray antennas [7,8,9,10] based on metasurfaces possess the advantages of high gain, low mass and simple feed. Compared with the reflectarray antenna, transmitarray antenna eliminates the shortcoming of feed blockage. However, the focal length of transmitarray antenna increases its own volume.

Folded transmitarray antenna (FTA) [11,12,13] based on path multiple reflection provides a feasible solution to obtain low profile. Nevertheless, it’s a challenge for FTA to achieve wideband operation. Moreover, the choice of feed antenna is also a critical factor for FTA design. Undoubtedly, choosing a wideband horn antenna will inevitably increase the volume of FTA. Alternatively, the co-planar integrated design of feed antenna can realize compact structure and reduce the profile of FTA. However, the bandwidth of planar antenna is usually limited. Therefore, there are few compact FTAs in the published literature. It is pivotal to design an efficient FTA with integrated wideband planar feed antenna.

Circularly polarized electromagnetic wave has good characteristics of anti-Faraday rotation and anti-interference [14,15], and is widely used in satellite communication system. Circularly polarized folded transmitarray antenna (CPFTA) [16,17] can meet the requirements of high gain, light weight and compact structure. It has great potential applications in satellite communication and point-to-point communication. Compared with FTA, the design difficulty of CPFTA increases significantly.

Stealth technology has attracted the attention of many countries. Radar cross section (RCS) is an important criterion to evaluate stealth performance. RCS reduction of antenna has always been a research hotspot [18,19,20]. Furthermore, it is meaningful to improve the radiation and scattering performance of the antenna simultaneously [21,22,23]. CPFTA has great potential applications in military fields, so it is valuable to design it with the integrated control of radiation and scattering. To the author’s knowledge, there is no published literature on the integrated radiation and scattering performance of CPFTA. We aim to design a high-gain, low-profile, and low-RCS transmitarray antenna, which has application in mobile satellite communication.

In this paper, the novel circularly polarized folded transmitarray antenna (NCPFTA) with integrated radiation and scattering performance is proposed and successfully proved by experiments. Compared with general circularly polarized folded transmitarray antenna (GCPFTA), the novel design can improve the axis ratio (AR) bandwidth significantly and reduce RCS simultaneously without adding any additional structure. The NCPFTA has the advantages of broadband, high gain, planar structure, low profile, convenience in installation and low RCS.

## 2. The Principle and Components of CPFTA

### 2.1. Principle of CPFTA

**Figure 1:**
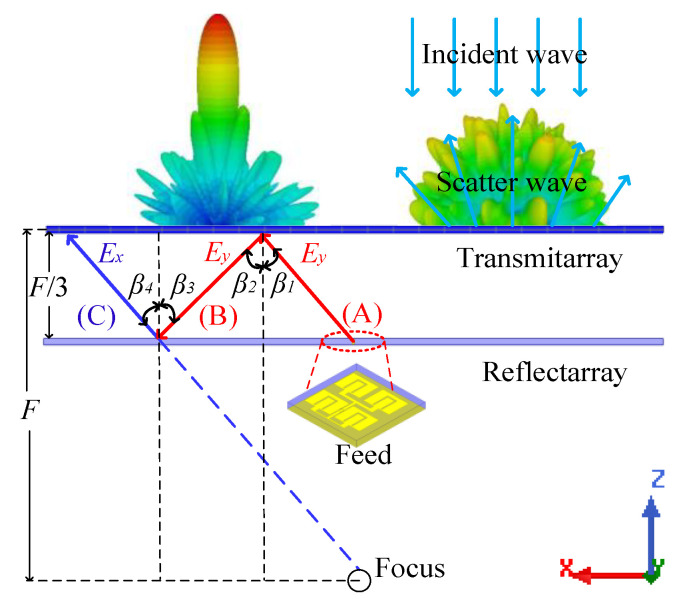
The configuration of the CPFTA in side-view.

Figure 1 illustrates the configuration of the CPFTA, which consists of linearly polarized feed antenna, reflectarray and transmitarray. The planar reflectarray and transmitarray are placed in parallel. The reflectarray can realize the cross-polarized reflection. For different linearly polarized incident waves, the transmitarray can realize dual different functions—co-polarized reflection and circularly polarized transmission.

In Figure 1, folded electromagnetic wave path is presented to illustrate the principle of CPFTA.

(1) Wave path A: the feed antenna radiates *y-*polarized waves at incident angle *β_1_*.
(2) Wave path B: the *y*-polarized waves are reflected by the transmitarray at angle *β_2_*.
(3) Wave path C: the *y-*polarized waves are converted into *x*-polarized waves by the reflectarray at angle *β_4_*.
(4) Finally, the transmitarray compensates the phase of *x-*polarized spherical waves and radiates circularly polarized quasi-plane waves.

Based on Fresnel reflection law, the incident angle equals to the reflection angle, i.e., *β_1_* = *β_2_* or *β_3_* = *β_4_*. According to the geometrical relationship shown in Figure 1, we can further derive that *β_1_* = *β_2_* = *β_3_* = *β_4_*. Therefore, the total path after two reflections is equivalent to the path from the focus (F) and the profile of FTA is reduced to F/3.

### 2.2. The Unit Cell of Transmitarray

**Figure 2:**
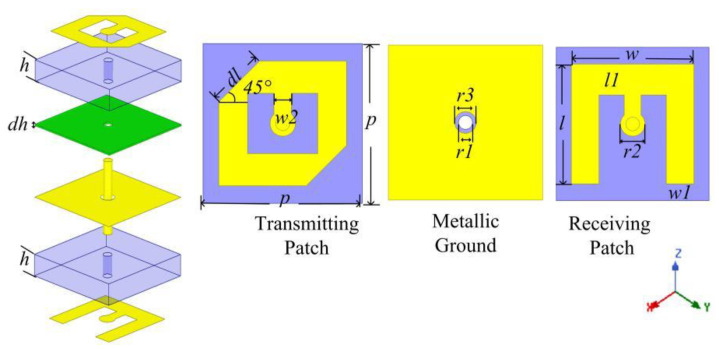
The schematic geometry of transmitarray unit cell.

The receiver-transmitter structure is adopted to construct transmitarray. The schematic geometry of the unit cell is shown in Figure 2. The unit contains three metallic layers separated by two identical substrates (TACONIC-TLX-8). The substrates are bonded together with ROGST-4450F. The top and bottom metallic layers are connected by metallic via. The middle layer is a metallic ground etched with circular hole. The periodicity (*p*) of the unit cell is 10 mm (about 0.33 λ at 10 GHz) and the profile of the unit cell is 3.1 mm (about 0.1 λ at 10 GHz). The parameters are tabulated in Table 1.

The structure of the unit cell can be regarded as back-to-back combination of two microstrip antennas. The bottom metallic layer is a E-shaped patch used to be a linearly polarized receiving antenna. The top metallic layer is an anisotropic patch used to be a circularly polarized transmitting antenna. The transmitarray works as follows. Firstly, the bottom E-shaped patch receives the linearly polarized electromagnetic wave radiating from the feed antenna. Then, the electromagnetic energy is transferred to the top anisotropic patch by the metallic via. Finally, the top anisotropic patch radiates circularly polarized electromagnetic wave.

Under *x-*polarized incident waves, *T_xx_* and *T_yx_*, represent the components of co-polarized and cross-polarized transmission, respectively. Analogously, *R_xx_* and *R_yx_* represent the components of co-polarized and cross-polarized reflection. ∆*φ* represents the phase difference between *T_yx_* and *T_xx_*. Defining AR [24] of circularly polarized waves and transmission ratio (TR) of the unit cell as:

(1) $$\mathrm{AR}=\left|20log_{10}\tan\left[0.5arcsin\left(\frac{2T_{xx}T_{yx}}{T_{xx}^{2}+T_{yx}^{2}}sin\Delta \varphi \right)\right]\right|$$

(2) $$\mathrm{TR}=\frac{\left(T_{yx}{}^{2}+T_{xx}{}^{2}\right)}{\left(T_{yx}{}^{2}+T_{xx}{}^{2}+R_{xx}{}^{2}+R_{yx}{}^{2}\right)}$$

**Figure 3:**
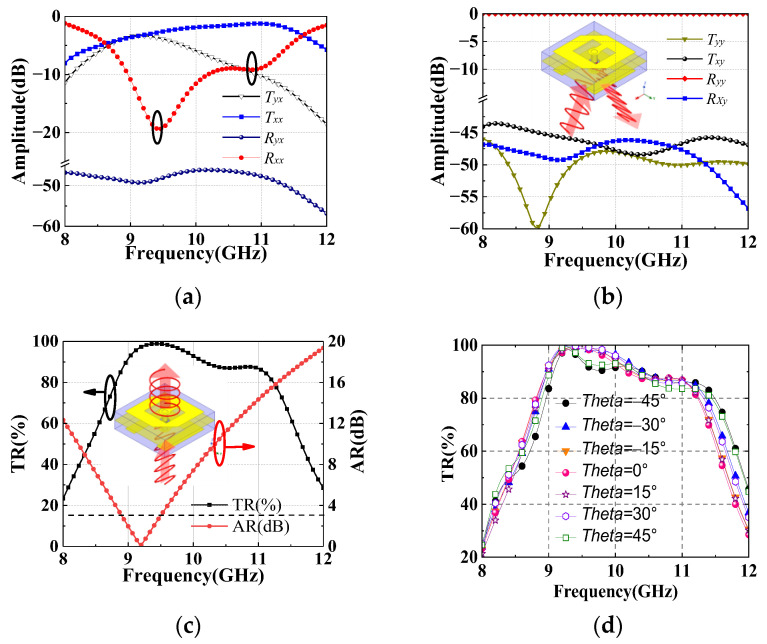
The reflection and transmission amplitudes of the unit cell under (**a**) *x-*polarized and (**b**) *y-*polarized incident waves. Simulated (**c**) AR and (**d**) TR under *x-*polarized incident waves.

Figure 3a plots the reflection and transmission amplitudes under *x-*polarized incident waves. Two obvious resonance frequencies of *R_xx_* are observed at 9.4 GHz and 10.8 GHz. The two resonance frequencies correspond to two TR peaks, as shown in Figure 3c. Around 9.2 GHz, the amplitude of *T_xx_* and *T_yx_* are equal, which satisfies the amplitude condition for generating circularly polarized waves. Similarly, *T_yy_*, *T_xy_*, *R_yy_* and *R_xy_* can be obtained under *y-*polarized incident waves. Since the polarization direction of incident waves is orthogonal to the E-shaped patch, the E-shaped patch hardly receives energy [25]. Hence, Figure 3b shows that the *y-*polarized incident waves are totally reflected.

The AR and TR are calculated under *x-*polarized incident waves, as shown in the Figure 3c,d. In the range of 8.9–9.5 GHz, the *x-*polarized incident waves are converted to circularly polarized transmission waves with the AR less than 3-dB. Furthermore, the TR of oblique incident waves is also studied. The simulated results show that the TR is larger than 80% in the range of 8.9–11.4 GHz with incident angle less than 45°.

Due to the feature of the receiver-transmitter structure, the top and bottom metallic layers are relatively independent. Hence, rotating the bottom E-shaped metallic patch with 180°can realize 1-bit transmission phase adjustment. This is the key for radiation beam focusing.

**Figure 4:**
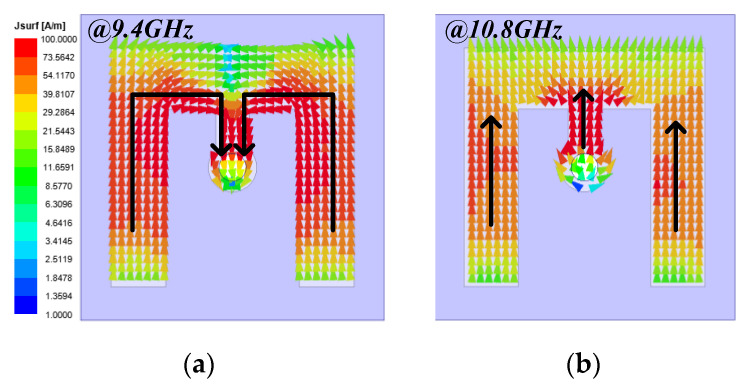
The current distributions on bottom E-shaped patch at (**a**) 9.4 GHz and (**b**) 10.8 GHz.

To better understand the working mechanism of the cell, the current distributions on bottom E-shaped patch at the two resonant frequencies are analyzed in Figure 4. At 9.4 GHz, the resonance is mainly formed by bended arms. Compared with Figure 4b, the current path is longer, and the current intensity is higher in Figure 4a. Therefore, the cell has lower amplitude of *R_xx_*, as seen in Figure 3a. While at 10.8 GHz, the direction of currents is consistent. Figure 4b shows that the E-shaped patch works such as ordinary microstrip antenna.

**Figure 5:**
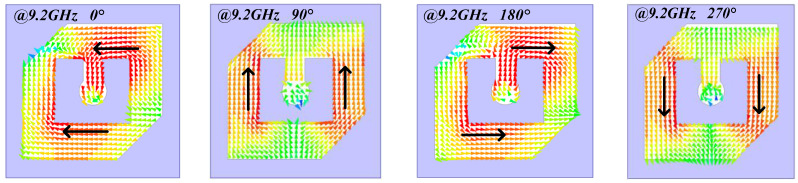
The current distributions on top anisotropic patch with phases of 0°, 90°, 180° and 270° at 9.2 GHz.

In addition, the current distributions of top anisotropic patch with phases of 0°, 90°, 180° and 270° are simulated at 9.2 GHz. Figure 5 depicts that the direction of current changes along the left-hand rotation. Therefore, the transmitarray radiates left-handed circularly polarized waves.

### 2.3. The Unit Cell of RA

**Figure 6:**
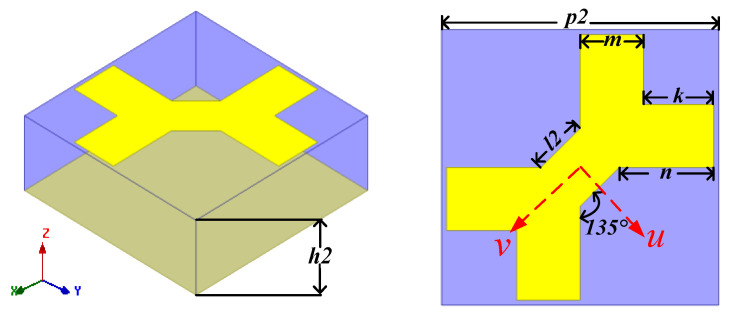
The schematic geometry of reflectarray unit cell.

The schematic geometry of reflectarray unit cell is shown in Figure 6. The unit is composed of top fractal metallic patch, substrate (TACONIC-TLX-8) and bottom metallic ground. The periodicity (*p2*) of the unit is 7 mm (about 0.23 λ at 10 GHz). Detailed structural parameters are *h2* = 2.5 mm (about 0.083 λ at 10 GHz), *m* = 1.6 mm, *l2* = 1.4 mm, *n* = 2.4 mm and *k* = 1.8 mm. The top fractal metallic patch is symmetrical along the *u*-axis and *v*-axis, respectively.

The reflectarray can realize cross-polarized reflection of linearly polarized incident waves. Defining polarization convert ratio (PCR) of cross-polarized reflection as:

(3) $$\mathrm{PCR}=\frac{{\left(R_{yx}\right)}^{2}}{\left[{\left(R_{xx}\right)}^{2}+{\left(R_{yx}\right)}^{2}\right]}$$

**Figure 7:**
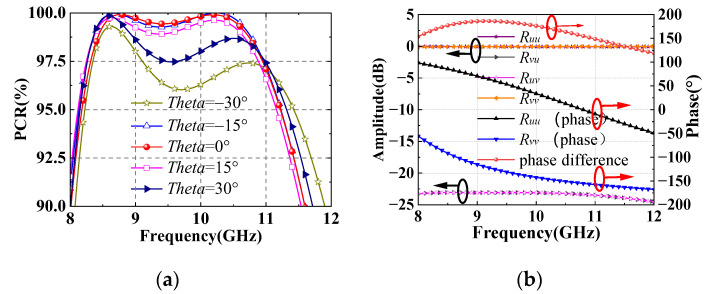
(**a**) The PCR of reflectarray with incident angle less than 30°. (**b**) The reflection amplitudes and phases in *u-v* coordinate.

Figure 7a shows that the PCR is more than 95% in the range of 8.2–11.2 GHz with incident angle less than 30°.

The principle of cell can be analyzed in *u*-*v* coordinate. Suppose that the *x-*polarized incident waves are decomposed into *u*-polarized and *v*-polarized waves in same phase. When the incident waves are *u*-polarized, *R_uu_* and *R_vu_* represent co-polarized and cross-polarized reflection, respectively. Similarly, *R_vv_* and *R_uv_* represent co-polarized and cross-polarized reflection of *v*-polarized incident waves. Since the cell is symmetric along the *u-v* coordinate, the *u*-polarized and *v*-polarized incident waves will realize co-polarized total reflection, as shown in Figure 7b. Moreover, the phase difference between the co-polarized reflection of *u*-polarized and *v*-polarized waves are around 180°. Therefore, the *u*-polarized and *v*-polarized reflected waves are combined into the *y-*polarized waves.

### 2.4. Planar Feed Antenna

**Figure 8:**
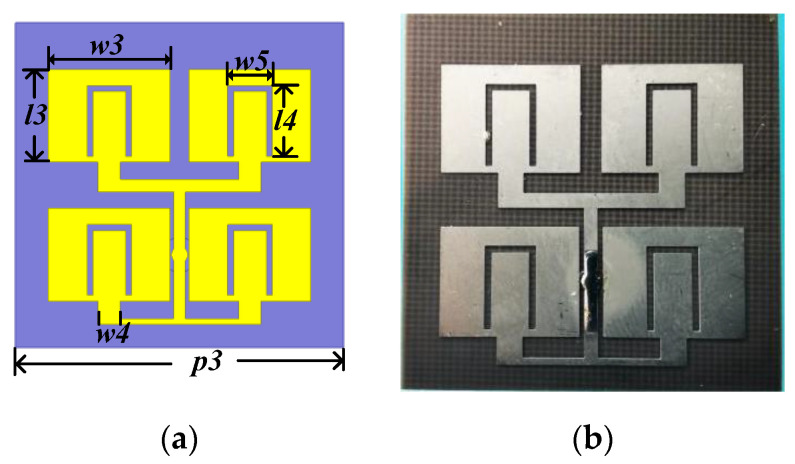
(**a**) The schematic geometry of planar feed antenna. (**b**) The fabricated planar feed antenna.

In order to integrate the feed antenna and reflectarray, a planar four element microstrip antenna with U-shaped gap is designed, as shown in Figure 8. The substrate is identical to the reflectarray. The detailed parameters of the feed antenna are as follows: *p3* = 14 mm, *w3* = 10.4 mm, *l3* = 8 mm, *w4* = 1.9 mm, *l4* = 6.1 mm, and *w5 =* 3.8 mm.

**Figure 9:**
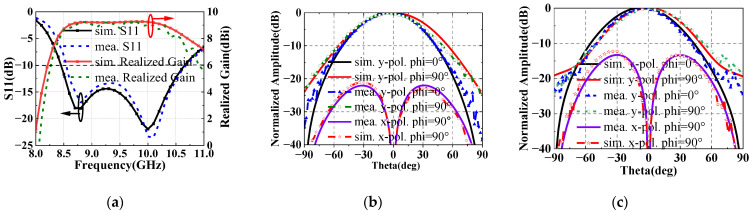
The simulated and measured (**a**) S11, realized gain, and radiation patterns at (**b**) 8.7 GHz and (**c**) 10 GHz.

Figure 9a depicts the reflection coefficients (S11) and realized gains of the proposed planar feed antenna. There are two resonant frequencies at 8.7 GHz and 10 GHz. The measured −10-dB S11 bandwidth is from 8.5 GHz to 10.7 GHz. The realized gain is stable in the operation bandwidth. The radiation patterns at the two resonant frequencies are plotted in Figure 9b,c, which are almost coincident within −10-dB beam width. The proximity of the first branch of the feeding line and the patch sides affects antenna radiation performance. As a result, the radiation patterns of 8.7 GHz and 10 GHz deviate from the broadside −5° on phi = 0° plane. The second branch of the feeding line cause the cross polarized component at phi = 90°, as shown in Figure 9b,c. The planar feed antenna has the advantage of broadband, low profile and easy integration.

## 3. The General Circularly Polarized Folded Transmitarray Antenna Design

### 3.1. Design of GCPFTA

**Figure 10:**
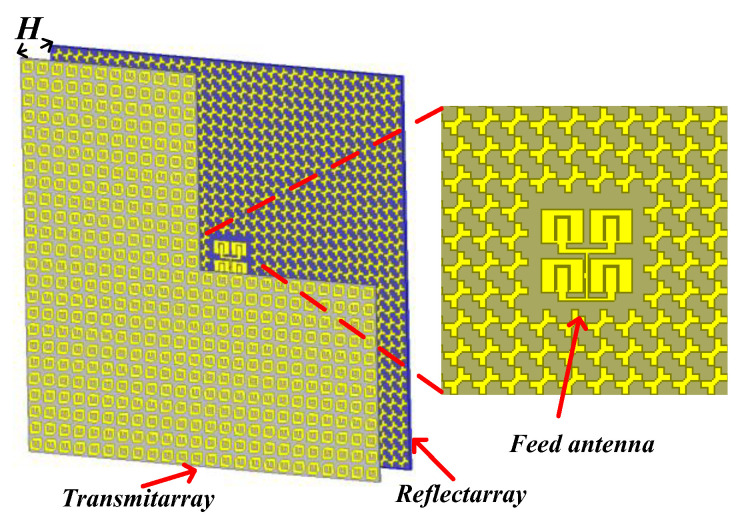
The schematic of GCPFTA.

The schematic of GCPFTA is shown in the Figure 10. To realize low profile, the ratio of focal length to aperture (F/D) of GCPFTA is selected as 0.45. The transmitarray contains 24 × 24 cells with total size of 240 mm × 240 mm × 3.1 mm (8 λ × 8 λ × 0.1 λ). Hence, the equivalent focal length (F) is 108 mm. According to the principle of FTA described in Section 2, the profile of FTA is reduced to F/3. Therefore, the height (*H*) between transmitarray and reflectarray is 36 mm.

The reflectarray contains 34 × 34 cells with total size of 238 mm × 238 mm × 2.5 mm (8 λ × 8 λ × 0.1 λ). 4 × 4 cells are replaced by planar feed antenna in the middle of reflectarray, as shown in Figure 10.

**Figure 11:**
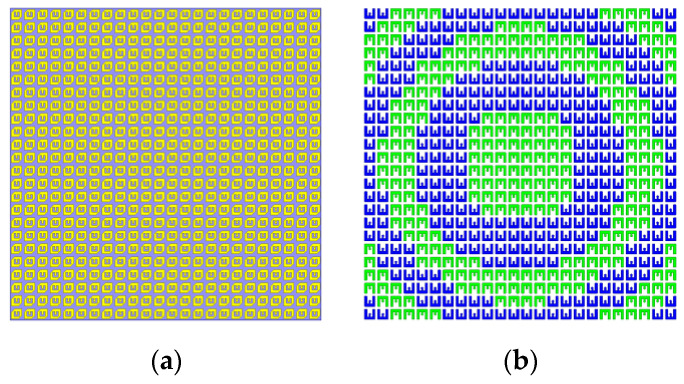
(**a**) The arrangement of 24 × 24 anisotropic patches on the top layer of transmitarray with size of 240 mm × 240 mm. (**b**) The arrangement of 24 × 24 E-shaped patches on the bottom layer of transmitarray with size of 240 mm × 240 mm.

The anisotropic patches on the top layer of the transmitarray are uniformly arranged, as shown is Figure 11a. As mentioned in Section 2, rotating the bottom E-shaped metallic patches of transmitarray can realize 1-bit transmission phase adjustment. *f*_0_ is the center working frequency of the feed antenna, and *c_0_* is light speed. (*m*, *n*) is used to represent the position of the transmitarray cell. The compensation phase of the unit (*m*, *n*) can be expressed as:

(4) $$\phi \left(m,n\right)=\frac{2\pi f_{0}}{c_{0}}\cdot \left(F_{mn}-F\right)$$

*F_mn_* is the distance from focus to cell (*m*, *n*), and *F* is the equivalent focal length. The compensation phase of the cell (*m*, *n*) can be discretized by the formula:

(5) $$C\left(m,n\right)=\{\begin{array}{c} \begin{array}{cc} 0^{\circ }, & -90^{\circ }<mod\left[\phi \left(m,n\right),360^{\circ }\right]\leq 90^{\circ } \end{array}\\ \begin{array}{cc} 180^{\circ }, & 90^{\circ }<mod\left[\phi \left(m,n\right),360^{\circ }\right]\leq 270^{\circ } \end{array} \end{array}$$

Consequently, the obtained arrangement of E-shaped patches on the bottom metallic layer is illustrated in Figure 11b.

**Figure 12:**
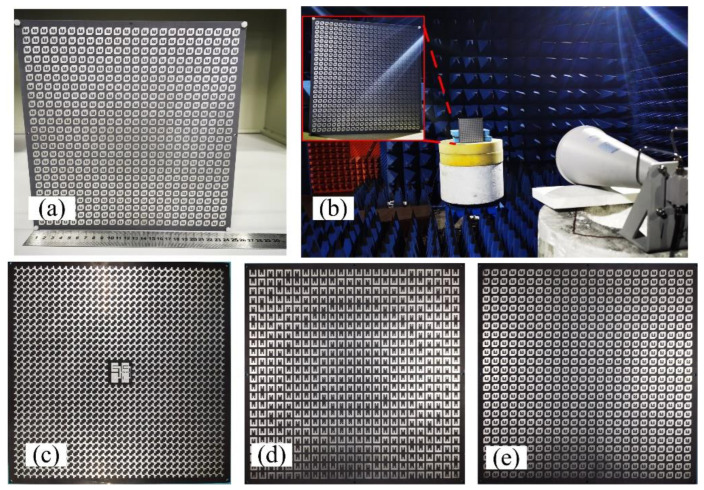
(**a**) The fabricated GCPFTA. (**b**) The measurement of GCPFTA in anechoic chamber. (**c**) The integrated reflectarray and planar feed antenna with total size of 238 mm × 238 mm × 2.5 mm. (**d**) The E-shaped patches on the bottom layer of transmitarray with size of 240 mm × 240 mm. (**e**) The anisotropic patches on the top layer of transmitarray with size of 240 mm × 240 mm.

The GCPFTA based on low-cost printed circuit board technique is fabricated and assembled, as shown in Figure 12. The height between transmitarray and reflectarray is controlled by dielectric cylinders. The measurement of GCPFTA is carried out in an anechoic chamber and the data was processed by averaging multiple measurements.

### 3.2. Simulated and Measured Results

**Figure 13:**
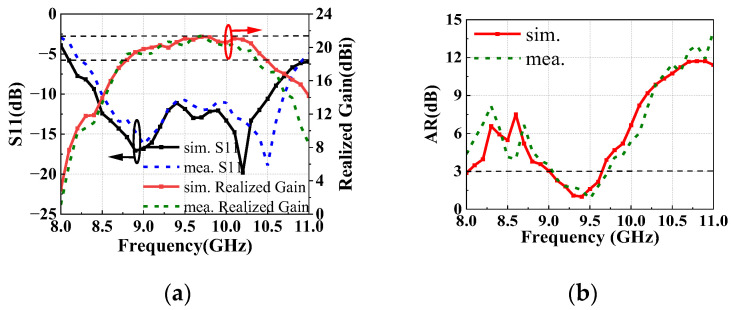
Simulated and measured results of GCPFTA (**a**) S11, realized gain and (**b**) AR.

The simulated and measured results of GCPFTA are shown in Figure 13. The GCPFTA obtains S11 bandwidth at range of 8.5–10.6 GHz and the 3-dB realized gain bandwidth of 8.8–10.3 GHz. Comparatively, the 3-dB AR bandwidth is narrow at range of 9.0–9.6 GHz, which is basically in accordance with the circularly polarized narrow bandwidth of transmitarray shown in Figure 3c. Ultimately, the narrow 3-dB AR bandwidth limits the working bandwidth of GCPFTA.

## 4. The Novel Circularly Polarized Folded Transmitarray Antenna Design

### 4.1. Design of NCPFTA

Generally, it’s a challenge to design wideband cell of transmitarray for CPFTA, since the transmitarray cell should possess four characteristics:

(1) High transmission ratio: the energy of the feed can be radiated through the transmitarray and the impedance match is acceptable.
(2) The manipulation of transmission phase: compensating the phase of the spatial path difference from feed antenna.
(3) Polarized conversion: linearly polarized incident waves are converted into circularly polarized transmission waves.
(4) Selectively polarized transmission: *x-*polarized waves can be transmitted while the *y-*polarized waves will be totally reflected.

To simplify the requirements for the cell of transmitarray, sequential rotation technique [26,27] is applied to design CPFTA for the first time. It is worth noting that the only difference between the proposed GCPFTA and NCPFTA is different arrangements of transmitarray. The novel design method can simplify the design difficulty significantly without adding any additional structure.

**Figure 14:**
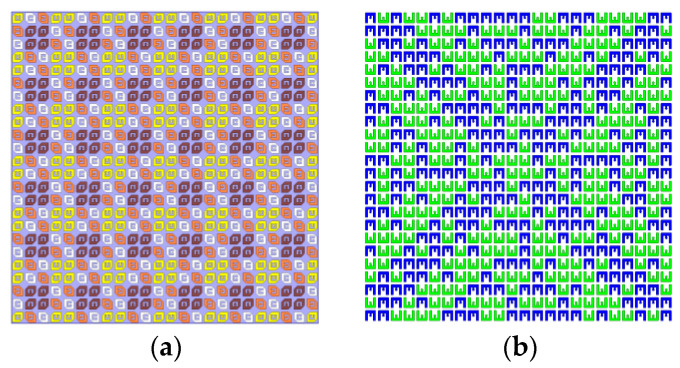
(**a**) The arrangement of 24 × 24 anisotropic patches with size of 240 mm × 240 mm. (**b**) The arrangement of 24 × 24 E-shaped patches with size of 240 mm × 240 mm.

2 × 2 sub-array second-order sequential rotation is adopted [28], and the anisotropic patches of the transmitarray are arranged as shown in the Figure 14a. In order to realize radiation beam focusing, both the compensation of spatial path difference and sequential rotation phase are considered simultaneously. Based on Formula (4), the phase compensation can be expressed as:

(6) $$\phi \left(m,n\right)=\frac{2\pi f_{0}}{c_{0}}\cdot \left(F_{mn}-F\right)+rotation_{mn}$$

**Figure 15:**
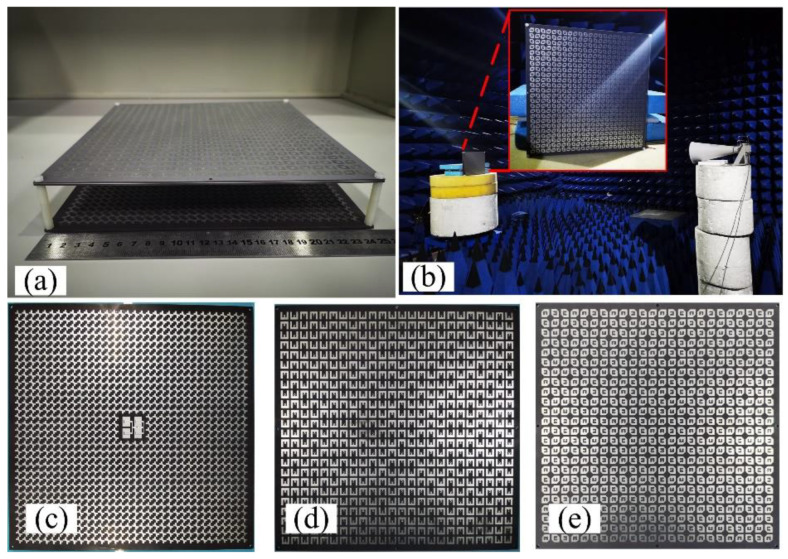
(**a**) The fabricated NCPFTA. (**b**) The measurement of NCPFTA in microwaves anechoic chamber. (**c**) The integrated reflectarray and planar feed with total size of 238 mm × 238 mm × 2.5 mm. (**d**) The bottom layer of transmitarray with size of 240 mm × 240 mm. (**e**) The top layer of transmitarray with size of 240 mm × 240 mm.

The *rotation_mn_* is the feeding phase of sequential rotation in unit (*m*, *n*). The bottom E-shaped patches of transmitarray are used for 1-bit phase compensation. The arrangement of E-shaped patch is shown in Figure 14b. Based on the above descriptions, NCPFTA is fabricated, assembled and tested, as illustrated in Figure 15.

### 4.2. Simulated and Measured Results

**Figure 16:**
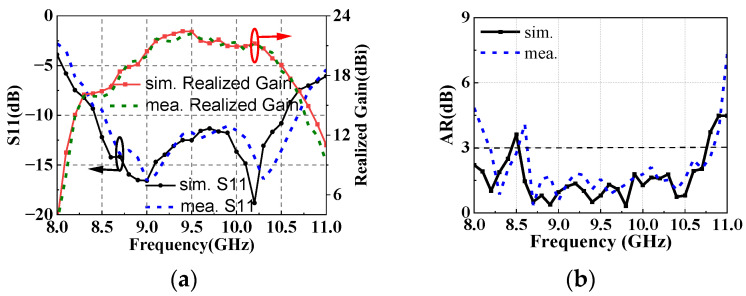
Simulated and measured results of NCPFTA (**a**) S11, realized gain and (**b**) AR.

The simulated and measured results of NCPFTA are shown in Figure 16. The results show that NCPFTA maintains S11 bandwidth at range of 8.5–10.6 GHz. The 3-dB realized gain bandwidth is from 8.9 GHz to 10.5 GHz (relative bandwidth 16.5%), and the peak gain is 22.4 dBi with the aperture efficiency 23.9%. Moreover, the 3-dB AR of NCPFTA shows ultra-wideband at range of 8.6–10.9 GHz (relative bandwidth 23.6%), which obviously proves the effectiveness of the proposed design method.

**Figure 17:**
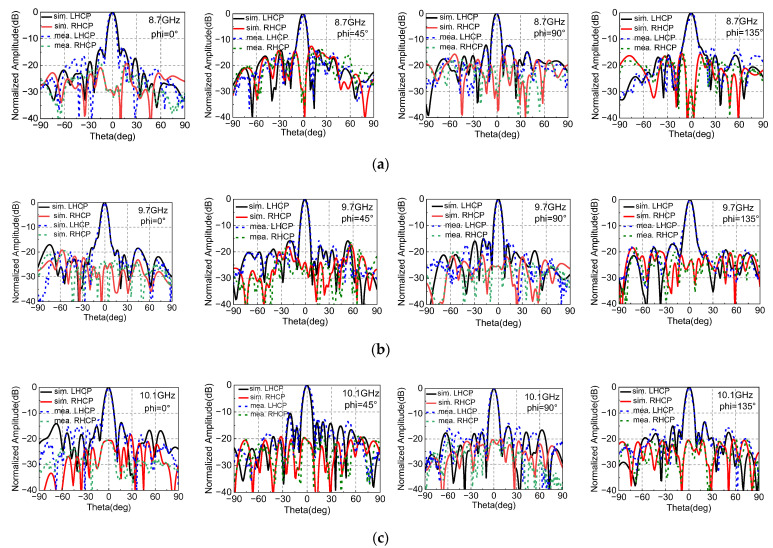
Simulated and measured radiation patterns in the phi = 0°, phi = 45°, phi = 90° and phi = 135° at (**a**) 8.7 GHz, (**b**) 9.7 GHz and (**c**) 10.1 GHz.

Figure 17 shows the simulated and measured radiation patterns of NCPFTA in phi = 0°, phi = 45°, phi = 90° and phi = 135°. The amplitude of left-hand circularly polarized (LHCP) and right-hand circularly polarized (RHCP) differ greatly in the direction of main beam. Stable high gain pencil-shaped radiation patterns are observed at 8.7 GHz, 9.7 GHz and 10.1 GHz. The measured results are in good agreements with the simulated results.

Compared with the ideal directivity of the aperture, multiple factors lead to gain loss of the fabricated NCPFTA. A detailed gain loss analysis is summarized in Table 2. The overall gain loss mainly comes from transmitarray, including 1-bit phase quantization loss, taper loss, transmission and reflection loss of transmitarray. Other losses are composed of fabrication loss, assembly and measurement error.

## 5. Performance Discussion

The integrated radiation and scattering performance of the NCPFTA are discussed in this section. The radiation performances of planar feed antenna, feed antenna with RA, GCPFTA and NCPFTA are compared. On the other hand, the scattering performances of GCPFTA, NCPFTA and the same size metallic board are analyzed.

### 5.1. Radiation Performance

**Figure 18:**
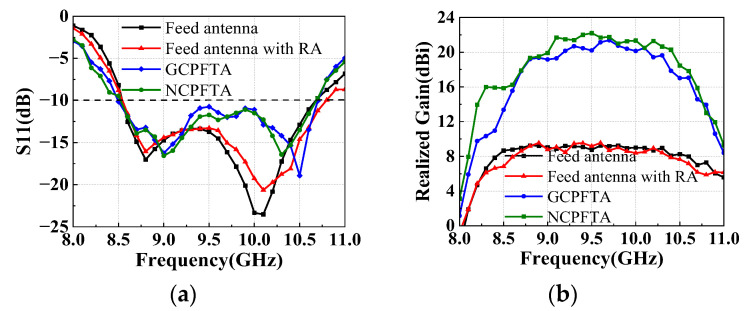
(**a**) The measured S11 of antennas. (**b**) The measured realized gain of antennas.

Figure 18a plots the measured S11 of four antennas. The impedance match of the planar feed antenna is slightly affected by the reflectarray and transmitarray. Both the GCPFTA and NCPFTA still maintain the broad band from 8.5 GHz to 10.6 GHz. Therefore, the broad band of the planar feed antenna and the well match of the transmitarray are proved.

Furthermore, the measured realized gain is compared and analyzed, as shown in Figure 18b. Compared with the planar feed antenna, the realized gain of the feed with reflectarray fluctuates slightly, which is caused by the coupling between the planar feed and the RA, as shown in Figure 10. Similarly, the realized gain of the GCPFTA and NCPFTA show slight fluctuations. 

Comparing the 3-dB AR of GCPFTA and NCPFTA, Figure 13b and Figure 17b show that the bandwidth of NCPFTA is greatly widen.

At present, there are little open literature focusing on the CPFTA. To further demonstrate the advantages of our work, some literature is selected for comparison, as listed in the Table 3 below. It can be seen that the proposed NCPFTA in this paper has appropriate radiation performance.

### 5.2. Scattering Performance

**Figure 19:**
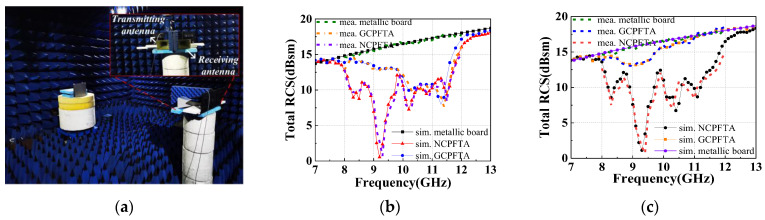
(**a**) The RCS measurement in microwaves anechoic chamber. The simulated and measured total RCS of the NCPFTA and the GCPFTA under (**b**) *x-*polarized and (**c**) *y-*polarized detection waves.

To the authors’ best knowledge, the scattering performance of CPFTA is studied for the first time in this paper. In order to make a comparison, the RCS of the same size metallic board is simulated and measured. Due to the limitation of experimental conditions, RCS at X-band was measured, as shown in Figure 19a. For the detection wave in the broadside direction, the NCPFTA presents the effect of diffuse scattering, as shown in Figure 1. The total RCS of the NCPFTA and GCPFTA in normal direction are shown in Figure 19b,c. Both under *x-*polarized and *y-*polarized detection waves, the NCPFTA has lower RCS in broad band. For *y-*polarized detection waves, the NCPFTA shows obvious RCS reduction at 7.7–12.7 GHz. Compared with GCPFTA, the maximum RCS reduction is 12.9 dB at 9.4GHz.

**Figure 20:**
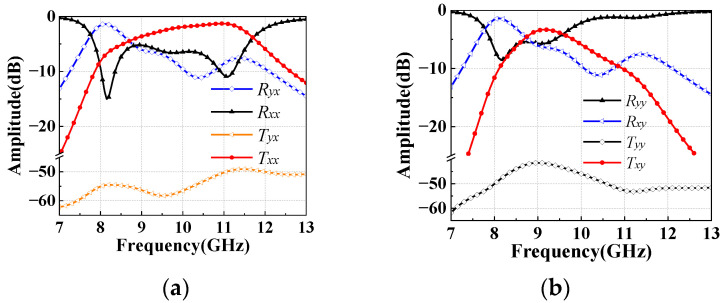
The amplitudes of co-polarized reflection, cross-polarized reflection, co-polarized transmission and cross-polarized transmission under (**a**) *x-*polarized and (**b**) *y-*polarized detection waves.

In order to further explore the reasons for RCS reduction, the cell of transmitarray is simulated under *x-*polarized and *y-*polarized detection waves. The amplitudes of reflection and transmission are shown in Figure 20. The transmitarray cells of GCPFTA are uniformly arranged, as shown in Figure 11a. In addition, the cell shows different electromagnetic responses to *x-*polarized and *y-*polarized detection waves, as shown in Figure 20. Hence, the total RCS of the GCPFTA is different obviously under the different polarized detection waves, as shown in Figure 19b,c. However, because of the sequential rotation technique of the NCPFTA, the cell arrangement of transmitarray is similar along the *x-*axis and *y-*axis, as seen in Figure 14a. Therefore, the total RCS of the NCPFTA is similar under *x-*polarized and *y-*polarized detection waves, as shown in Figure 19b,c.

For the GCPFTA, the frequency of high transmission amplitude is basically consistent with the frequency of small RCS, as shown in Figure 19 and Figure 20. Compared with metallic board, the reason for the RCS reduction is the transmission of detection waves. After penetrating the transmitarray, the detection waves are converted into cross-polarized waves by the reflectarray. Then, the waves are reflected many times in the air cavity between the transmitarray and reflectarray, and are dispersed to multiple directions.

For the NCPFTA, there are two reasons for RCS reduction. One is the transmission of detection waves. The other is the reflected phase cancellation of transmitarray. Figure 20 shows large reflection components. The anisotropic patches of transmitarray with different rotation directions will possess different reflection performances. The reflected waves can realize reflected phase cancellation between each other. Hence, the NCPFTA possess better stealth performance.

## 6. Conclusions

The sequential rotation technique is successfully applied to design a NCPFTA in this paper. The integrated radiation and scattering design of CPFTA is presented for the first time. Experimental results of NCPFTA show 3-dB realized gain bandwidth from 8.9 GHz to 10.5 GHz (relative bandwidth of 16.5%), and the peak gain is 22.4 dBi with the aperture efficiency 22.9%. Moreover, the 3-dB AR of NCPFTA shows ultra-wideband at range of 8.6–10.9 GHz (relative bandwidth 23.6%). Compared with GCPFTA, the NCPFTA possess better stealth performance. It is worth noting that the novel design method can simplify the design difficulty significantly without adding any additional structure. The proposed NCPFTA has potential application in mobile satellite communication.

## Figures and Tables

**Table 1:** Parameters of transmitarray unit cell.

**Parameter**	*w*	*l*	*l1*	*w1*	*r2*	*r1*
**Value (mm)**	8	7.8	2	1.8	1.4	0.9
**Parameter**	*r3*	*w2*	*dh*	*dl*	*h*	*p*
**Value (mm)**	1.4	1.1	0.1	3.6	1.5	10

**Table 2:** Gain loss analysis of the proposed NCPFTA.

Aperture Size	240 mm × 240 mm
Measured Gain	22.4 dBi (23.9%)
Ideal Directivity	28.6 dBi
Total Loss	6.2 dB
Spillover Loss	0.7 dB
Taper Loss	0.6 dB
Transmission Loss of TA	1.0 dB
Reflection Loss of TA	0.2 dB
Reflection Loss of RA	0.3 dB
Other Losses	0.6 dB
Phase quantization Loss	2.8 dB

**Table 3:** Comparison between the proposed CPFTA and some literature.

Ref.	Type	Pol.	Feed Type	Feed Integration	H/D	Center Fre. (GHz)	Peak Gain (Dbi)	S11 BW (%)	3-dB AR BW (%)	3-dB Gain BW (%)	RCS Analysis
[11]	FTA	LP	horn	NO	0.3	27.5	25.2	21.6	——	18	NO
[12]	FTA	LP	horn	NO	0.33	21	21.9	>9.5	——	6.7	NO
[16]	FTA	CP	Microstrip	YES	0.16	10.3	21.8	16.3	23.2	11.6	NO
[29]	FRT	CP	Array	YES	0.29	5.3	22.8	15.1	>11.3	13	NO
GCPFTA	FTA	CP	Microstrip	YES	0.15	9.5	21.4	21.1	6.5	15.7	YES
NCPFTA	FTA	CP	Microstrip	YES	0.15	9.5	22.4	21.1	23.6	16.5	YES

## Data Availability

The data presented in this study are available on request from the corresponding author.
